# Characterisation of Lipoma-Preferred Partner as a Novel Mechanotransducer in Vascular Smooth Muscle Cells

**DOI:** 10.3390/cells12182315

**Published:** 2023-09-19

**Authors:** Alexandra Sporkova, Taslima Nahar, Mingsi Cao, Subhajit Ghosh, Carla Sens-Albert, Prisca Amayi Patricia Friede, Anika Nagel, Jaafar Al-Hasani, Markus Hecker

**Affiliations:** Department of Cardiovascular Physiology, Heidelberg University, Im Neuenheimer Feld 326, 69120 Heidelberg, Germany; alexandra.sporkova@physiologie.uni-heidelberg.de (A.S.);

**Keywords:** VSMC, LPP, mechanotransduction, vascular remodelling, mechanosensitive genes

## Abstract

In arteries and arterioles, a chronic increase in blood pressure raises wall tension. This continuous biomechanical strain causes a change in gene expression in vascular smooth muscle cells (VSMCs) that may lead to pathological changes. Here we have characterised the functional properties of lipoma-preferred partner (LPP), a Lin11–Isl1–Mec3 (LIM)-domain protein, which is most closely related to the mechanotransducer zyxin but selectively expressed by smooth muscle cells, including VSMCs in adult mice. VSMCs isolated from the aorta of LPP knockout (LPP-KO) mice displayed a higher rate of proliferation than their wildtype (WT) counterparts, and when cultured as three-dimensional spheroids, they revealed a higher expression of the proliferation marker Ki 67 and showed greater invasion into a collagen gel. Accordingly, the gelatinase activity was increased in LPP-KO but not WT spheroids. The LPP-KO spheroids adhering to the collagen gel responded with decreased contraction to potassium chloride. The relaxation response to caffeine and norepinephrine was also smaller in the LPP-KO spheroids than in their WT counterparts. The overexpression of zyxin in LPP-KO VSMCs resulted in a reversal to a more quiescent differentiated phenotype. In native VSMCs, i.e., in isolated perfused segments of the mesenteric artery (MA), the contractile responses of LPP-KO segments to potassium chloride, phenylephrine or endothelin-1 did not vary from those in isolated perfused WT segments. In contrast, the myogenic response of LPP-KO MA segments was significantly attenuated while zyxin-deficient MA segments displayed a normal myogenic response. We propose that LPP, which we found to be expressed solely in the medial layer of different arteries from adult mice, may play an important role in controlling the quiescent contractile phenotype of VSMCs.

## 1. Introduction

Blood vessels dynamically respond to local hemodynamic forces and signalling molecules to control organ perfusion and blood pressure. While vasomotor tone is determined by the contractile state of vascular smooth muscle cells (VSMCs) and reflects acute adaptations of the vessel diameter to changes in (blood) pressure and vasoactive mediators, the response to chronic hemodynamic stress is very complex and involves structural adaptations of the blood vessels. VSMCs possess a high degree of cellular plasticity that allows them to alter their phenotype in response to extracellular signals and cues, including chronic increases in blood pressure, to maintain vascular homeostasis [1]. Under physiological conditions, VSMCs exhibit a differentiated contractile phenotype characterized by a low rate of proliferation [2]. Upon vascular injury, in atherosclerosis or hypertension, VSMCs re-enter the cell cycle and become dedifferentiated, assuming a “synthetic” phenotype with enhanced proliferation and migration as well as an increased synthesis of extracellular matrix (ECM) proteins [1]. This activated phenotype is associated with the increased expression of remodelling-associated signalling molecules, including cell cycle regulators, mitogen-activated protein kinases (MAPK) and matrix metalloproteinases (MMP) as well as a reduction in VSMC-specific markers, including calponin, smooth muscle-myosin heavy chain (SM-MHC) and α-smooth muscle actin (α-SMA) [3]. This plasticity of VSMCs allows the blood vessels to structurally adapt; however, if the healing process fails to resolve appropriately, it can drive cardiovascular pathologies, including hypertension, atherosclerosis, intimal hyperplasia, restenosis and aneurysm formation [3,4]. Therefore, a better understanding of the pivotal mechanisms by which VSMCs sense and integrate the mechanical cues from the ECM that initiate their phenotypic dedifferentiation and reinforce maladaptive remodelling processes could benefit the development of new treatment strategies for vascular diseases.

VSMC–ECM interactions are mediated by focal adhesions (FAs), the main cellular network for mechanotransduction at which multiple proteins, the ECM, integrins and cytoskeletal proteins interact [5]. Each VSMC possesses multiple FAs, providing a basis for the mechanical properties of blood vessels because they play a crucial role in regulating the intrinsic tone and stiffness of the VSMCs. About 100 proteins have been shown to be associated with FAs, and among those, LIM-domain containing proteins exhibit a highly conserved mechanism in transducing mechanical signals from the cytoskeleton to other cellular compartments including the nucleus [6,7]. Zyxin is an important LIM-domain protein acting as a mechanotransducer most notably in endothelial cells in which it regulates the expression of many mechanosensitive genes [8,9]. In VSMCs, it is also involved in the biomechanical response to force [10].

LPP is structurally the closest relative to zyxin, sharing about 41% of its protein sequence homology. Like zyxin, it is associated with FAs, binds actin stress fibres and possesses transactivation capacity in the nucleus [11]. LPP competes with zyxin for the same binding site at filamentous α-actin and plays a regulatory role in cytoskeletal organization, cell adhesion and migration during vascular injury [12,13]. In vitro studies indicate that LPP may be able to compensate for the loss of zyxin in VSMCs, thereby maintaining the quiescent contractile phenotype by supporting the expression of smooth-muscle-specific genes [14]. LPP is highly enriched in the medial layer of arterial blood vessels and considered a smooth-muscle-cell-specific gene based on in vivo expression studies [12]. Given its emerging role as an important regulator of VSMC architecture, adhesion and migration, we investigated the intriguing hypothesis that LPP is an important mechanotransducer in VSMCs of both conduit and resistance-sized arteries. We show that unlike zyxin, LPP is highly enriched in the medial layer of arteries but not in the endothelium. Our data indicate that both LIM-domain proteins stabilize the quiescent contractile phenotype of VSMCs in vitro. Interestingly, only the lack of LPP in arteries produces a vascular phenotype characterized by an attenuated myogenic response to increased intravascular pressure.

## 2. Methods

### 2.1. Isolation of VSMCs from Mouse Aorta

Cryopreserved heterozygous LPP knockout (LPP-KO) embryos on a mixed 129/SvJ-C57BL/6J background were kindly provided by Professor Wim Van de Ven at the Department of Human Genetics, KU Leuven, Belgium. They were transferred to pseudopregnant C57BL6/J foster mothers, and the resulting offspring were backcrossed onto the C57BL6/J background at least ten times. Wildtype (WT) mice referred to herein represent littermates derived from the breeding of heterozygous LPP-KO mice.

For preparing VSMCs from these mice, the descending aorta starting at the outlet of the right renal artery was dissected and washed twice in calcium-free Dulbecco’s PBS. The aorta was then briefly (15 min) pre-digested in collagenase II-containing DMEM (1 mg/mL, Worthington Biochemical, Lakewood, NJ, USA) to easily peel off the adventitial layer. Subsequently, the aorta was cut in 1 mm rings and digested in collagenase II (1 mg/mL) and elastase (0.15 mg/mL) for approximately 3 h, followed by cell dispersion. VSMCs were cultured in DMEM supplemented with 15% foetal bovine serum (FBS) plus penicillin/streptomycin and fungizone at 37 °C with 5% CO_2_. Cells in passages 3–5 were used in experiments.

### 2.2. Immunocytochemistry

Cells were fixed with 4% paraformaldehyde (PFA) and blocked with casein solution (0.25% casein, 0.1% BSA, 15 mmol/L NaN_3_ and 50 mmol/L Tris at a pH of 7.6). After fixation, cells were incubated at ambient temperature with primary antibodies at the following concentrations (rabbit anti-zyxin, HPA004835, rabbit anti-LPP, HPA017342; both diluted at a ratio of 1:75) and incubated overnight at 4 °C. Alpha-smooth muscle actin (α-SMA, F3777) staining was performed at a dilution of 1:200. After rinsing, cells were incubated with secondary antibodies for 2 h at ambient temperature followed by 10 min with 4′,6-diamidino-2-phenylindole (DAPI) (Invitrogen via Thermo Fisher Scientific, Karlsruhe, Germany) in PBS to counterstain the nuclei and then mounted in Mowiol (Calbiochem via Merck-Millipore, Darmstadt, Germany).

For staining of the blood vessels, femoral and MA segments were fixed in 4% paraformaldehyde, dehydrated, embedded in paraffin and cut into 3 μm thick sections. Antigens were retrieved by incubating re-hydrated tissue sections with citrate buffer (pH 6.0) at 100 °C for 15 min. The sections were then incubated with blocking solution and the same procedure as for immunostaining of the cells was applied. For confocal microscopy, an IX81 microscope equipped with an IX-DSU disk unit and the MT20 multi-wavelength illumination system was used in combination with the cellSens software package (version 1.12, Olympus Deutschland, Hamburg, Germany).

### 2.3. Application of Cyclic Stretch

An FX-5000 tension system (Flexcell via Dunn Labortechnik, Asbach, Germany) was used to subject the cells to 13% cell elongation at 0.5 Hz. The cells were exposed to cyclic stretch for 1, 8 or 24 h to study the dynamics of LPP distribution in response to biomechanical stretch.

### 2.4. Cell Proliferation Assay

Proliferation was determined by counting the cells at two different time points, at the beginning after seeding and 72 h after seeding. Briefly, mouse VSMCs were seeded into 6-well plates at a density of 20,000 cells/well. After 6 h, cells in one plate were fixed with 4% PFA to determine the number of cells at the starting point (t = 0). The remaining cells seeded on the second plate were further grown and fixed after 72 h (t = 72 h). The fixed cells were stained with DAPI, 6 random images of the individual wells were analysed by counting the cells per field of view and the proliferation rate was determined.

Cells grown in 3D spheroids were also used to analyse the rate of proliferation. To this end, the spheroids (see below) were embedded in paraffin, and 5 µm thick sections were prepared and stained for the proliferation marker Ki67.

### 2.5. Generation of 3D Spheroids

Murine aortic VSMCs were detached with trypsin, centrifuged at 1000 rpm for 5 min and counted by using an automated cell counter (CASY, OMNI Life Science, Bremen, Germany). For each spheroid, droplets of 25 µL containing 500 cells in culture medium with 0.24% (*w/v*) methyl cellulose (Sigma-Aldrich/Merck, Darmstadt, Germany) plus 15% (*v/v*) FBS were pipetted onto squared petri dishes (approximately 100 spheroids per dish) that were placed upside down in the incubator to generate hanging drops and cultivated for 24 h.

For the **collagen gel invasion assay**, the spheroids were resuspended into collagen matrices. A total of 4.5 mL acidic collagen extract of rat tails was mixed with 500 µL of 10× M199 medium (M0605, Sigma-Aldrich) and quickly titrated with 0.2 M NaOH to neutralize the solution. The spheroids were quickly resuspended into the collagen solution, and the resultant mixture was pipetted into pre-warmed 24-well plates (0.5 mL spheroid/collagen gel suspension pro well). After collagen gel polymerization, 100 μL of DMEM medium (containing 15% FBS) was added on top of the gel surface.

As previously described [15], the spheroid angiogenesis assay was used to measure the invasion of VSMC spheroids into the collagen gel. The images of the spheroids were taken after 24 h at 10× magnification, and the cumulative length and number of sprouts originating from individual spheroids were measured. Analysis was performed using the cellSens Dimension software (version 1.9, Olympus).

For the **spheroid contraction assay**, spheroids were also generated by the hanging drop method as described above and harvested in cell culture medium with 15% FCS, but they were plated on top of the collagen gel matrices, employing a recently established protocol [16]. Briefly, for the preparation of collagen gels, all ingredients were held on ice. Collagen gel stock solution (4.5 mL with 2 mg/mL type I collagen) was carefully mixed with 500 µL of 10× M199 and quickly neutralized by adding approximately 175 µL sterile NaOH (0.2 M, solution turning from yellow to light pink) to facilitate the polymerization of the collagen gel. Subsequently, the same volume (5 mL) of DMEM with 15% FBS was added, thoroughly mixed and quickly pipetted at a volume of 0.5 mL into the wells of a 24-well plate. The plates containing the gels were placed into an incubator to enhance gel polymerization. Then, the harvested spheroids were plated at a density of 15–20 spheroids per well.

### 2.6. Gelatinase Assay

MMP activity was measured in the slices from the 3D spheroids by using the DQ-gelatine assay (Thermo Fisher Scientific). The zinc-fixed paraffin-embedded sections were incubated with 100 µM DQ gelatine in the reaction buffer at 37 °C for 2 h. Nuclei were counterstained with DAPI. Sections were examined by fluorescence microscopy.

### 2.7. Transfection of Cells

As published previously [14], the zyxin expression plasmid was constructed by subcloning a full-length polymerase chain reaction fragment (PCR) including the first stop codon (position 305 to 1999; NM 011777) derived from VSMC cDNA into the cDNA 6.2/N-EmGFP TOPO 5.9-kb vector using the TOPO cloning reaction according to the manufacturer’s instructions (TOPO Mammalian Expression Vector Kit, Invitrogen). The same principle was applied for the LPP expression plasmid that encompassed the full-length PCR fragment with the first stop codon (position 551 to 2392, NM 178665.5). A GFP-expressing construct (Lonza Bioscience via Biozym Scientific, Hessisch Oldendorf, Germany) was used as a control for all transient transfection experiments. Nucleofector^TM^ technology (Lonza Bioscience) was used for transient transfection of the expression plasmids into the cultured VSMCs according to the manufacturer’s instructions. The transgenic expression of LPP and zyxin was confirmed by immunofluorescence and Western blot analyses. Cells were analysed 48 h post transfection.

### 2.8. Vascular Myography

All animal studies were performed with permission of the Regional Council Karlsruhe and in conformance with the Guide for the Care and Use of Laboratory Animals published by the US National Institutes of Health (NIH publication No. 85-23, revised 1996). After sacrificing the mice by cervical dislocation, the mesenteric arcade was removed from the mice and placed into physiological salt solution (PSS) with the following composition (in mM): 140 NaCl, 5 KCl, 1.8 CaCl_2_, 1 MgCl_2_, 10 HEPES and 10 glucose at a pH of 7.4. MAs of 3rd or 4th order were carefully cleaned from fat and surrounding connective tissue followed by mounting of the vessels on glass micropipettes in the chamber of the myograph (DMT 110P). Vessels were equilibrated at 37 °C while superfused with PSS. After equilibration, the response to 10 μM phenylephrine (PE) was tested. Only vessels responding with >30% constriction to PE and showing a development of myogenic tone at 80 mm Hg were used in further measurements. To assess the myogenic reactivity of the vessels, with starting intravascular pressure of 20 mm Hg, pressure was gradually increased in 20 mm Hg steps. MA started to respond with myogenic constriction at 60–80 mm Hg. Pressure was increased only when the vascular diameter was stable, usually every 10 min until a final pressure of 160 mm Hg was reached. The same pressure–response curve was then performed in Ca^2+^-free buffer in the presence of EGTA to obtain the values for the passive pressure diameter curve. Myogenic tone was then calculated as follows: myogenic tone (%) = (passive diameter − active diameter/passive diameter) × 100. Concentration–response curves to agonists were performed at an intravascular pressure of 80 mm Hg in a cumulative manner. Distensibility of the MA segments (distention index) was expressed as the ratio of the diameter at 120 mm Hg to the diameter at 20 mm Hg.

### 2.9. Statistical Analysis

Data were analysed by GraphPad Prism version 10.0.2 (GraphaPad Software) and the results are represented as mean ± SD of individual experiments with VSMCs isolated from individual mice. In the spheroid contraction assay, more than 5 technical replicates were included to calculate the means of the contractile/dilatory responses for each treatment group including more than 4 individual preparations. For the analysis of differences between two experimental groups, unpaired Student’s *t*-test was used for which *p* ˂ 0.05 was considered statistically significant. For the analysis of differences between three or more experimental groups, one-way analysis of variance followed by Šídák’s multiple comparisons test for selected pairs of groups were used. Data from the vascular experiments are presented as mean ± SEM of n samples, i.e., vessel segments isolated from individual mice. For the dose–response curves, EC_50_ and E_max_ were calculated from individual experiments. The area under the curve (AUC) was calculated for the individual pressure–response curves. Differences between two individual groups as well as between three or more individual groups were analysed as described above with *p* < 0.05 considered statistically significant.

## 3. Results

### 3.1. Sub-Cellular Distribution of LPP in VSMCs

**Figure 1:**
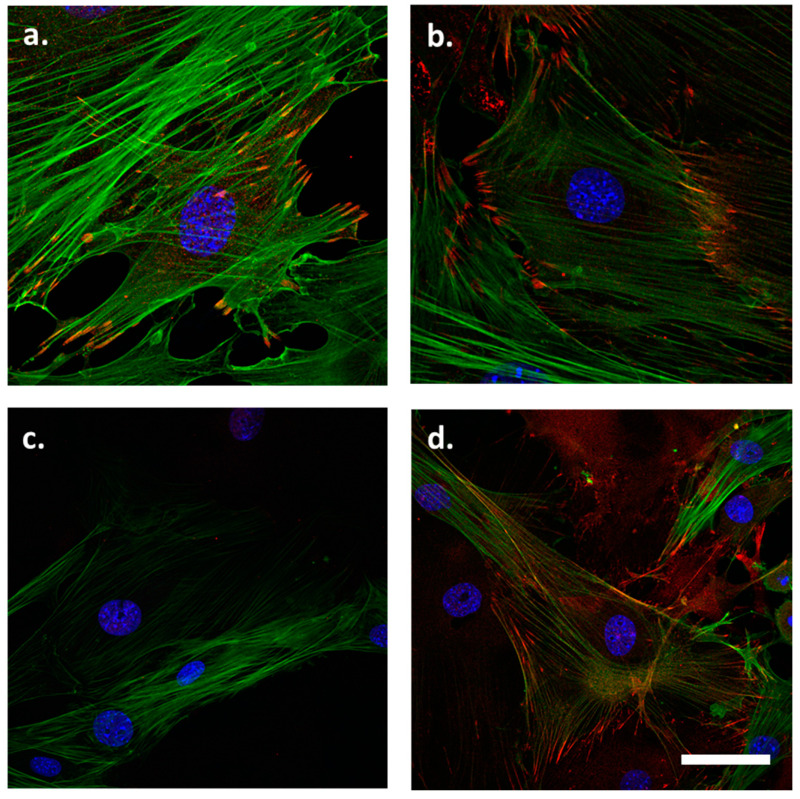
Distribution of (**a**) LPP and (**b**) zyxin in cultured VSMCs isolated from the aorta of 3-month-old C57BL/6 mice. LPP and zyxin immunoreactivity is shown in red, and stress fibres stained with an anti-α-SMA antibody are shown in green. Nuclei were counterstained with DAPI (blue). (**c**) LPP staining in LPP-KO VSMCs to control for the specificity of the antibody, and (**d**) zyxin staining in the LPP-KO VSMCs (**d**). Representative images; the scale bar shown in d represents 20 µm.

In the VSMCs isolated from 3-month-old WT mice, LPP was localized to the FAs and in particular to the tips of the stress fibres (Figure 1a). Zyxin was also localized to these sites with a somewhat stronger preference for the FAs (Figure 1b). The abundance of both proteins in the nucleus was rather low, if they were present at all. Figure 1c confirms the absence of LPP in the LPP-KO VSMCs while the abundance and distribution of zyxin did not differ between LPP-KO and WT VSMCs (Figure 1d).

### 3.2. Distribution of LPP after Cyclic Stretch

**Figure 2:**
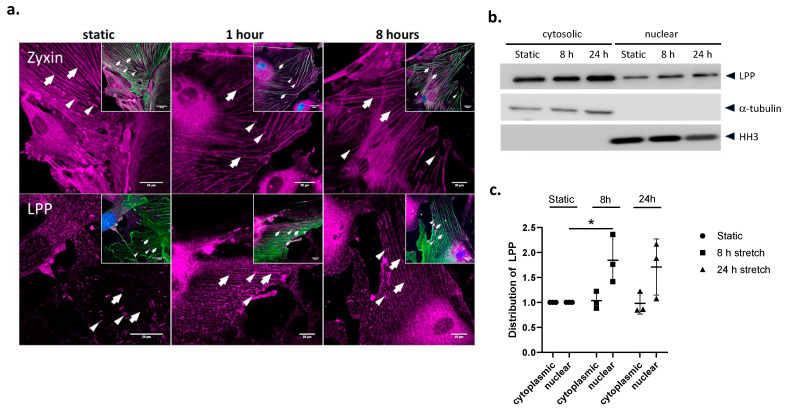
Subcellular distribution of zyxin and LPP in cultured VSMCs (WT) in vitro. (**a**) Indirect immunofluorescence confocal laser scanning images of VSCM cultured for 48 h under static conditions (static), followed by 1 h or 8 h of exposure to cyclic stretching (13% elongation, 0.5 Hz). Inserts: Overlay of zyxin- or LPP-specific (purple) and α-smooth muscle actin (SMA)-specific (green) antibody staining; nuclei are additionally stained with DAPI (blue). Narrow arrow heads: focal adhesions, arrows: (position of) SMA-containing fibres; scale bar: 20 μm. (**b**) Representative Western blot analysis of cytosolic and nuclear fractions of VSMCs from 3-month-old WT mice pre-cultured for 48 h followed by 24 h incubation under static conditions or 8 and 24 h of exposure to cyclic stretching (13% elongation, 0.5 Hz), respectively. Alpha-tubulin and histone H3 (HH3) served as indicators for the purity of the subcellular fractions and as loading controls. (**c**) Statistical summary of 3 independent experiments with individual preparations of VSMCs isolated from the aorta of the 3-month-old WT mice. * *p* ˂ 0.05 as indicated.

To test whether the sub-cellular distribution of LPP is affected by mechanical strain, the WT VSMCs were exposed to 13% cyclic stretching for 1 and 8 h, respectively (Figure 2a). In response to 1 h of stretching, LPP was more intensely and evenly localized to the stress fibres of the VSMCs. The concentrated staining of LPP at the FAs as seen in the static VSMCs was less intense in the stretched cells due to redistribution of LPP in response to the mechanical stimulus. In contrast, zyxin was localized to the stress fibres already under static conditions and this sub-cellular distribution did not change much after 1 or 8 h of stretching. There was no apparent translocation of zyxin or LPP to the nucleus of the stretched VSMCs after 1 or 8 h of exposure to cyclic stretching (Figure 2a). An additional Western blot analysis of the cytoplasmic and nuclear fraction of these cells revealed a moderate (approximately 1.7-fold) increase in the abundance of LPP in the nucleus following 8 and 24 h of exposure to cyclic stretching (Figure 2b,c). This nuclear translocation of LPP was less pronounced than that of zyxin (not shown) and collectively was too weak to be visualized by high-resolution laser scanning confocal microscopy.

### 3.3. Lack of LPP and its Effect on VSMC Proliferation

**Figure 3:**
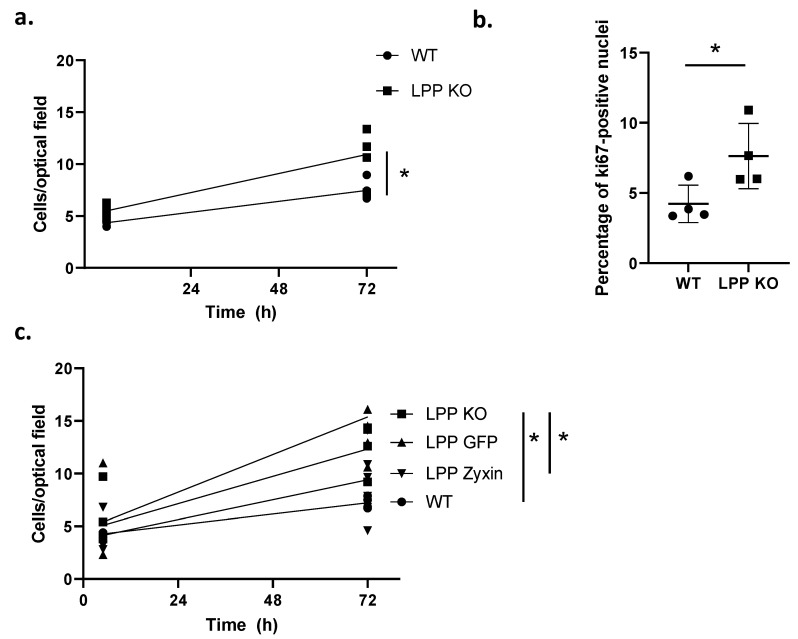
Proliferation of WT and LPP-KO VSMCs. (**a**) The rate of proliferation was determined by counting the number of cells per optical field of view at 0 and 72 h. Each data point represents an individual VSMC’s preparation with >6 separate optical fields of view analysed. n = 4, * *p* ˂ 0.05 as indicated. (**b**) Percentage of WT and LPP-KO VSMCs grown in 3D spheroids expressing the proliferation marker Ki67. n = 4, * *p* < 0.05 as indicated. (**c**) Transient overexpression of zyxin and eGFP in LPP-KO VSMCs and their effect on the rate of proliferation. Each data point represents an individual VSMC’s preparation with >6 fields of view analysed. n = 5, * *p* < 0.05 as indicated.

To characterize the phenotype of the LPP-KO VSMCs, their rate of proliferation under 2D culture conditions 6 and 72 h post seeding was compared to that of their WT counterparts. While there was a 2.5-fold increase in the number of cells in the LPP-KO group, the WT VSMCs only showed a 1.7-fold increase in cell number over this period (Figure 3a), indicating a slower proliferation rate than the LPP-KO VSMCs. We further compared the proliferation rate of VSMCs grown in 3D spheroids that closely mimic the natural environment of VSMCs in the blood vessel wall. Employing the proliferation marker Ki-67, we observed that twice as many LPP-KO VSMCs were positively stained in the nucleus compared to their WT counterparts (Figure 3b).

Additionally, we performed a bromodeoxyuridine (BrdU) incorporation assay to assess the rate of cell proliferation in a second, alternative assay. While the LPP-KO VSMCs incorporated BrdU at an increased rate, the overall difference between the groups was not significant (cf. Figure S1). The lesser difference in the proliferation rate in this assay may be due to the lack of proper penetration of BrdU into the cells, resulting in a non-uniform concentration of BrdU in the cells, limiting our observation of the effect size. Collectively, this data show that LPP-KO VSMCs proliferate faster than their WT counterparts, suggesting a shift towards the activated synthetic phenotype.

To investigate whether the overexpression of zyxin in LPP-KO VSMCs can compensate for the loss of LPP and thereby retransform them to the quiescent contractile phenotype, we transiently transfected LPP-KO VSMCs with a zyxin expression construct and monitored their rate of proliferation. The overexpression of zyxin resulted in about a three-fold increase in zyxin levels according to the Western blot analysis (cf. Figure S2), and while the non-transfected cells again showed the highest rate of proliferation (Figure 3c), this was normalized to below the level of the GFP-transfected control VSMCs upon zyxin overexpression.

### 3.4. D-Migration of LPP-Deficient VSMCs

**Figure 4:**
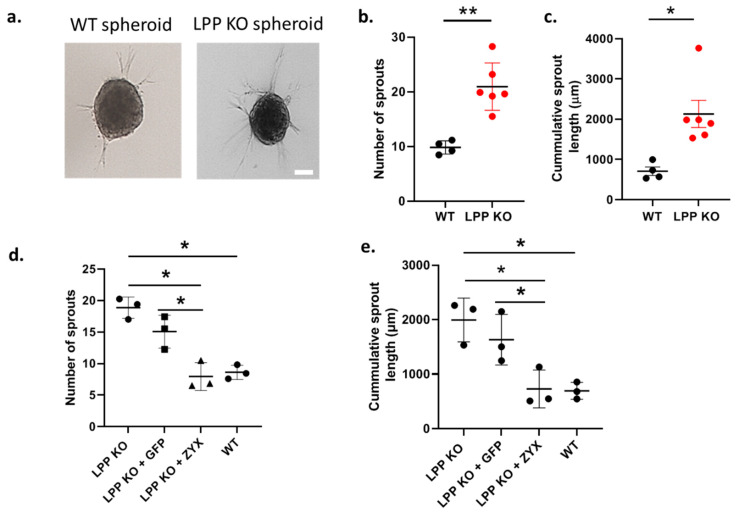
LPP-KO VSMCs and WT VSMCs grown in 3D spheroids embedded in collagen gels form sprouts and invade the collagen gel. (**a**) Representative images, the scale bar represents 50 µm. (**b**) Summary of the average number of sprouts produced by individual LPP KO VSMC (n = 6, in red) and WT VSMC preparations (n = 4) with >10 spheroids per VSMC preparation analysed using the cellSens software. (**c**) Summary of the cumulative distance travelled by individual sprouts of a VSMC spheroid. n = 6 and n = 4 as in (**b**), * *p* < 0.05, ** *p* < 0.01 as indicated in (**b**,**c**) Transient overexpression of zyxin and eGFP in LPP-KO VSMCs and their effect on (**d**) the average number of sprouts and (**e**) cumulative sprout length. Each data point represents an individual VSMC preparation with >10 spheroids per VSMC preparation analysed. n = 3, * *p* ˂ 0.05 as indicated.

We further studied the degree of sprouting of VSMC spheroids embedded in a collagen gel as a measure of their migration capacity (invasion by cellular sprouts into the collagen gel) in a 3D environment (Figure 4a). LPP-KO VSMC spheroids produced an approximately two-fold greater number of sprouts as compared to their WT counterparts (Figure 4b). The cumulative sprout length of the LPP-KO VSMC spheroids was also significantly increased when compared to the WT VSMC spheroids (Figure 4c). Again, these results point to the activated synthetic phenotype of the LPP-KO VSMCs.

Moreover, the overexpression of zyxin but not that of the control construct GFP in the LPP KO VSMC spheroids significantly reduced both the number of sprouts formed and the cumulative sprout length (Figure 4d,e), suggesting that zyxin may compensate for the lack of LPP in regulating the VSMC phenotype.

### 3.5. Gelatinase Activity of LPP-KO VSMCs in 3D Culture

We next investigated whether LPP-deficient VSMCs grown as 3D spheroids exhibit a greater ECM-remodelling capacity that could facilitate their invasion and migratory behaviour as observed in the collagen gels. To this end, we employed the DQ gelatine assay to determine the proteolytic activity of MMP-2 and MMP-9, two major matrix metalloproteinases that are released from activated synthetic VSMCs [17]. Our data suggest that the degradation of DQ gelatine was significantly more pronounced in the LPP-KO VSMC spheroids as compared to their WT counterparts (cf. Figure S3).

### 3.6. Functional Characteristics of LPP-Deficient VSMC Spheroids

**Figure 5:**
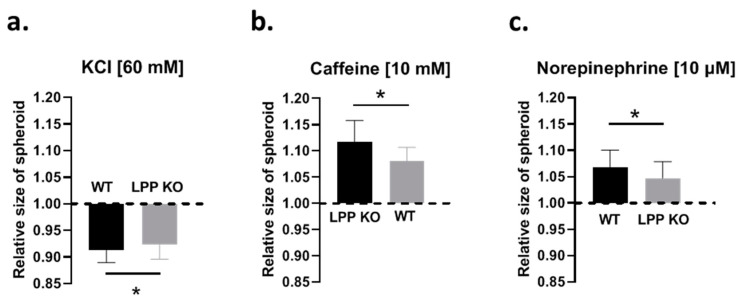
Comparison of the contractile properties of the VSMCs grown as 3D spheroids attached to a collagen gel. (**a**) The contractile response of the VSMC spheroids to potassium chloride (KCl, 60 mM) is calculated as reduction in the area of the collagen gel covered by the spheroids. (**b**) Relaxation response of the VSMC spheroids to caffeine (10 mM) and to norepinephrine (NE, 10 μM) (**c**). Each column represents the average responses of VSMC spheroids with >5 spheroids analysed per preparation and a total of 6 VSMCs preparations from individual animals per group (LPP KO or WT). * *p* ˂ 0.05 as indicated.

To study whether the loss of LPP affects the contractile and dilatory capacity of the VSMCs in a 3D environment, we employed a spheroid contraction assay [16]. After testing several contractile agents, we opted for potassium chloride (KCl), which, by way of depolarization and hence activation of voltage-dependent L-type calcium channels (LTCC, not shown), produced a stable reproducible contraction of VSMCs grown in spheroids. Contraction to KCl (60 mM) occurred immediately after its application and ranged from 6 to 10% (which presented as a reduction in the collagen gel surface area covered by the spheroid). As shown in Figure 5a, the contractile response of the LPP-KO VSMC spheroids was significantly smaller than that of their WT counterparts. When the spheroids were only treated with the vehicle, the area covered by the spheroid was not changed (not shown). Response to KCl was significantly inhibited, but not blocked by nifedipine (10 μM). We subsequently pre-treated the spheroids with caffeine (10 mM for 10 min) to deplete intracellular calcium stores. In the caffeine pre-treated spheroids, nifedipine abolished the response to KCl (cf. Figure S4).

The application of caffeine alone (10 mM) did not produce a contraction as might have been expected by the initial Ca^2+^ release from the intracellular calcium stores. Instead, caffeine initiated a slow relaxation, peaking at about 3 min, with an increase in the ‘size’ of the VSMC spheroids ranging from 5 to 14%. The LPP-KO VSMC spheroids reacted with a significantly attenuated functional response to caffeine as compared to that of their WT counterparts (Figure 5b). While caffeine enhances the Ca^2+^ sensitivity of the ryanodine receptors that become activated by basal intracellular Ca^2+^ levels, resulting in the release and depletion of intracellular Ca^2+^ stores, caffeine also promotes vasodilation by acting as an inhibitor of phosphodiesterase activity [18]. In addition, caffeine inhibits myosin light chain kinase (MLCK) and actin–myosin interactions [19,20] and thereby promotes dilation, as we observed in the VSMC spheroids.

Furthermore, we applied norepinephrine (NE, 10 μM), which also resulted in the relaxation of the VSMC spheroids but to a smaller degree than their response to caffeine. Like their response to caffeine, the effect of NE was smaller in the LPP-KO VSMC spheroids as compared to that of their WT counterparts (Figure 5c). Their relaxation to NE was abolished by the β-adrenergic antagonist propranolol (cf. Figure S4).

### 3.7. Expression of LPP and Zyxin in Blood Vessels Isolated from Adult Mice

**Figure 6:**
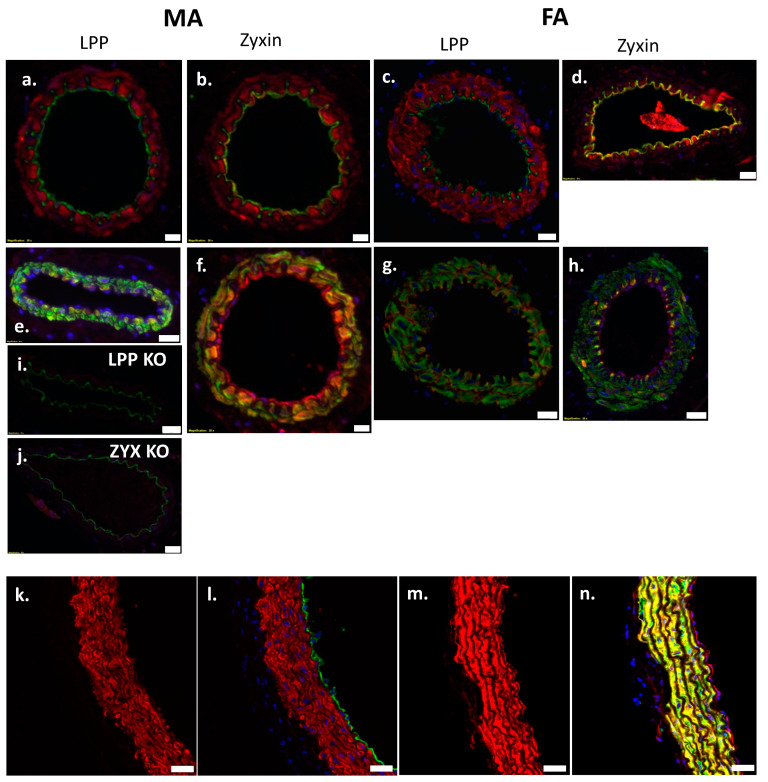
Immunofluorescence analysis of LPP and zyxin in 3rd-order mesenteric artery (MA) and femoral artery (FA) segments isolated from WT, LPP-KO or ZXY-KO mice. (**a**–**d**) Representative images for LPP ((**a**,**c**); red) and zyxin ((**b**,**d**); red) in MA and FA segments isolated from WT mice. To properly localize both LIM domain proteins, an anti-α-SMA antibody was used to stain the VSMCs in the media ((**e**–**h**), green) and an anti-CD31 antibody was used to visualize the endothelial cells lining the inner lumen of these segments ((**a**–**d**), green). While there is a clear colocalization of zyxin with the endothelium (**b**,**d**), LPP solely colocalizes with the medial VSMCs (**e**,**f**) with which zyxin also colocalizes but to a smaller extent. Staining of both LIM domain proteins in MA segments isolated from the respective knockout mice confirmed the specificity of the antibodies used (**i**,**j**). LPP ((**k**), red) and zyxin ((**m**), red) were also stained in segments of the aorta isolated from WT mice. Here, co-staining of LPP and CD31 ((**l**), green) confirmed their mutually exclusive localization, whereas co-staining of zyxin and α-SMA ((**n**), green) revealed that zyxin is highly abundant in the media of this large conduit artery (yellow colour indicates prominent colocalization with the VSMCs in the media) as well as a distinct staining of the endothelium. Scale bars represent 10 μm in (**a**–**c**,**f**) and 20 μm in (**d**,**e**,**g**–**n**).

To assess the potential functional role of LPP in different arterial blood vessels, we employed immunofluorescence analysis to assess the abundance of LPP or zyxin in the third- to fourth-order branches of the mouse MA that are considered as true resistance-sized arteries. Additionally, the femoral artery and aorta as two typical representatives of conduit arteries were analysed. Figure 6a shows the distribution of LPP in the mouse MA where LPP is confined to the medial layer co-localizing with α-SMA (Figure 6e). In contrast, LPP did not co-localize with the endothelial cell marker CD31 in the MA branches (Figure 6a). Virtually identical data were obtained for the femoral artery (Figure 6c,g). The staining of blood vessels isolated from the LPP-KO mice confirmed the specificity of the LPP antibody used, as these vessels revealed no positive staining for LPP (Figure 6i). While zyxin was also localized to the medial VSMC layer of the MA branches (Figure 6b) and the femoral artery (Figure 6d), where it co-localized with α-SMA (Figure 6f,h), zyxin was also unambiguously detected in the endothelium of these blood vessels (Figure 6b,d). No zyxin-positive staining was detected in the zyxin-deficient mice, validating the specificity of the zyxin antibody (Figure 6j). The immunofluorescence analysis of the specimens from the aorta revealed a similar distribution of LPP and zyxin as seen in the smaller-calibre arteries. LPP was detected solely in the media of the aorta (and not in the endothelium; Figure 6k,l show that the green CD31 fluorescence does not overlap with the red LPP staining), whereas zyxin was again clearly detected in the endothelium (Figure 6n) and in the media, overlapping with α-SMA staining (Figure 6m).

A similar conclusion for the differential expression of LPP in the arterial vessel wall can be derived from single-cell RNA sequencing analyses of lung and brain microvascular cells in which LPP was predominantly expressed in VSMCs and at a higher level than zyxin (cf. Figure S5), http://betsholtzlab.org/VascularSingleCells/database.html (accessed on 8 February 2023) [21,22].

### 3.8. MA Segments from LPP-KO Mice Exhibit Normal Contractile Responses to Adrenergic Stimulation, Potassium Chloride and Endothelin-1

**Figure 7:**
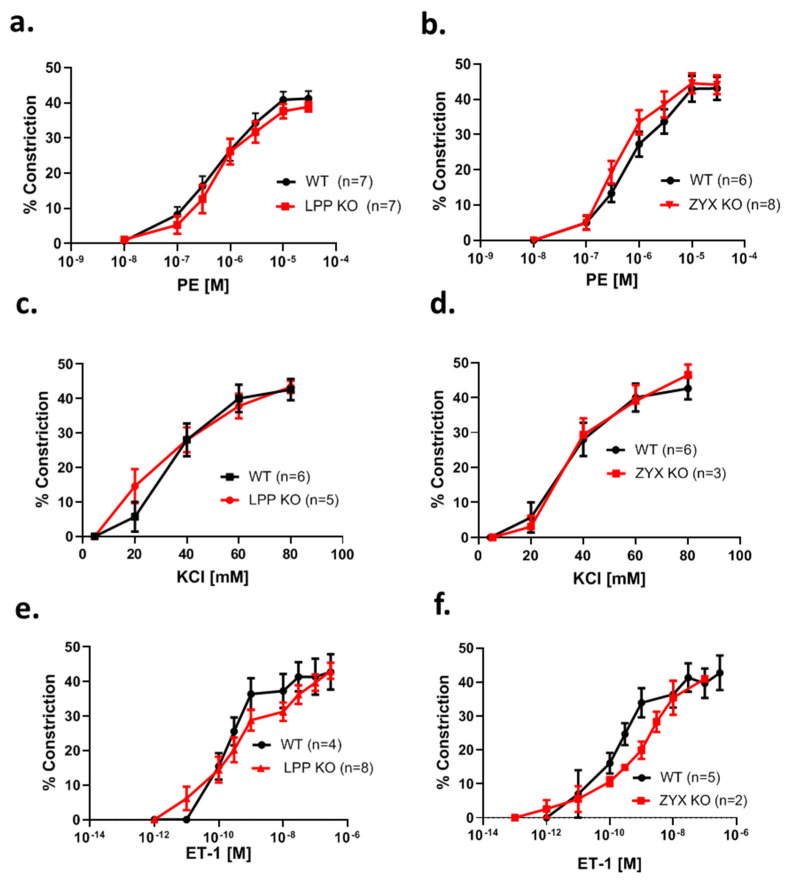
Comparison of the contractile response of 3rd-order mesenteric artery segments isolated from WT, LPP-KO or ZYX-KO mice to different vasoactive stimuli. The number of isolated perfused segments derived from individual mice is indicated on the graphs. Cumulative concentration–response curves for (**a**,**b**) phenylephrine (PE), (**c**,**d**) potassium chloride (KCl) and (**e**,**f**) endothelin-1 (ET-1).

To determine whether resistance-sized arteries isolated from LPP knockout mice exhibit normal contractile responses to vasoactive agonists, first a concentration–response curve for the α_1_-adrenoreceptor agonist phenylephrine (PE) was established. The MA segments from the LPP-KO mice responded to PE in a concentration-dependent manner with a similar EC_50_ value and the same maximum response as their WT counterparts (Figure 7a). Similar results were obtained with MA segments isolated from zyxin-deficient mice (ZYX-KO) (Figure 7b). We further employed KCl to depolarize the native VSMCs. As shown in Figure 7c, there was no difference in the magnitude of the responses to KCl between MA segments isolated from the WT or LPP-KO mice. Similarly, the vasoconstrictor response of the MA segments derived from the zyxin-KO mice to KCl was not different as compared to that of their WT counterparts (Figure 7d). Finally, we established a concentration–response curve to endothelin-1 (ET-1), which was not different between the LPP-KO and WT MA segments (Figure 7e). The same held true for the ZYX-KO and WT MA segments (Figure 7f).

### 3.9. The Myogenic Response Is Attenuated in LPP-Deficient Isolated Perfused MA Segments

**Figure 8:**
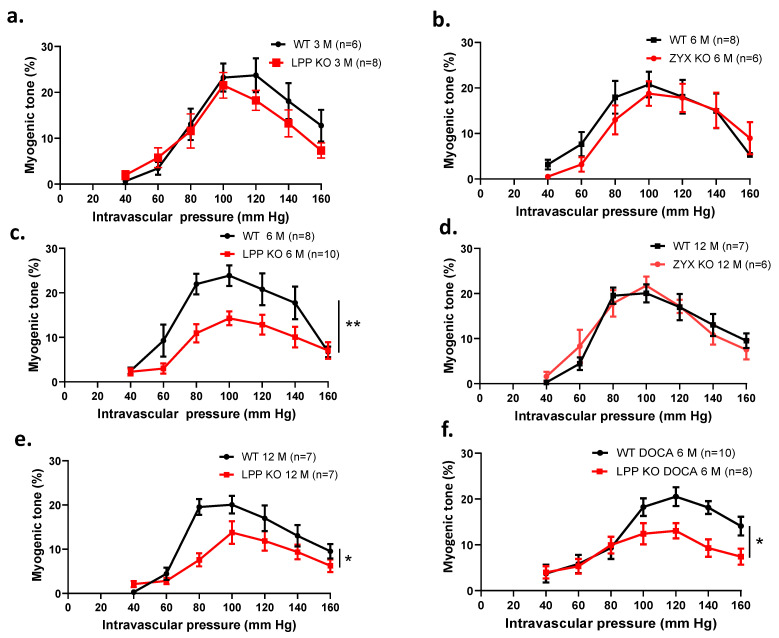
Comparison of the myogenic responses of 3rd-order mesenteric artery segments isolated from WT, LPP-KO or ZYX-KO mice. The number of isolated perfused segments derived from individual mice is indicated on the graphs. (**a**) Pressure–response curves for segments of (**a**) 3-month-old, (**c**) 6-month-old and (**e**) 12-month-old WT and LPP-KO mice; (**b**) 6-month-old and (**d**) 12-month-old WT and ZYX-KO mice; and (**f**) 6-month-old WT and LPP-KO mice made hypertensive using the DOCA-salt model of experimental hypertension. The area under the curve (AUC) was calculated for each individual group. * *p* ˂ 0.05, ** *p* ˂ 0.01 as indicated.

We further investigated the effect of raising the perfusion pressure and thus eliciting a myogenic response in the isolated perfused MA segments. Figure 8a shows the myogenic responses of MA segments isolated from 3-month-old LPP-KO mice. Both the LPP-KO and WT MA segments responded to a stepwise increase in intravascular pressure with active constriction, reaching a maximum response at 100 mm Hg. This myogenic tone was maintained at 120 mm Hg and declined thereafter. While there was no difference between the two types of segments at this age (Figure 8a), MA segments derived from 6-month-old LPP-KO mice revealed a strongly reduced myogenic response as compared to that of their WT counterparts (Figure 8c). Similarly, the myogenic response of MA segments isolated from 12-month-old (12 M) LPP-KO mice was significantly smaller as compared to that of MA segments isolated from age-matched WT mice (Figure 8e). In contrast, the myogenic tone in MA segments isolated from 6- or 12-month-old ZYX-KO mice was not different as compared to their WT counterparts (Figure 8b,d).

**Figure 9:**
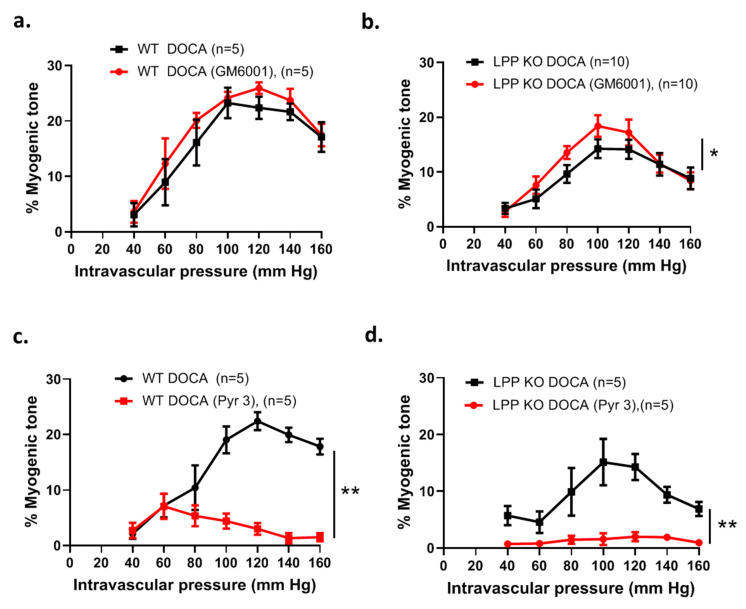
Susceptibility of the myogenic response of 3rd-order mesenteric artery segments isolated from WT or LPP-KO mice made hypertensive by employing the DOCA-salt model of experimental hypertension. The number of isolated perfused segments derived from individual mice is indicated on the graphs. Effect of the general gelatinase inhibitor GM6001 (0.5 µM) on (**a**) MA segments derived from (**a**) hypertensive WT and (**b**) LPP-KO mice and of the TRPC3 channel blocker Pyr 3 (3 µM) on MA segments isolated from (**c**) hypertensive WT and (**d**) LPP-KO mice. The area under the curve (AUC) was calculated for each individual group. * *p* ˂ 0.05, ** *p* ˂ 0.01 as indicated.

Moreover, the difference in the myogenic response between the MA segments isolated from the WT and LPP-KO mice was maintained when these were derived from 6-month-old (6 M) animals made hypertensive for 21 days using the deoxycorticosterone acetate (DOCA)-salt model of hypertension (Figure 9f), [23]. In MA segments isolated from 12-month-old hypertensive LPP-KO mice, the myogenic tone did not differ from the values measured in MA segments isolated from 6-month-old hypertensive LPP-KO mice (cf. Figure S6).

Since LPP-KO VSMCs present with a higher MMP activity (cf. Figure S3), we pre-treated the isolated perfused MA segments with the generic MMP inhibitor GM6001. While GM6001 did not affect the myogenic response of MA segments derived from hypertensive WT mice (Figure 9a), the myogenic response of MA segments isolated from hypertensive LPP-KO mice was significantly enhanced (Figure 9b).

To evaluate a possible mechanism responsible for the age-dependent attenuated myogenic response in the LPP-deficient MA segments, we employed Pyr3, a selective inhibitor of the transient receptor potential canonical 3 (TRPC3) channel-mediated influx of extracellular Ca^2+^ [24]. While Pyr3 significantly diminished the myogenic response of MA segments derived from hypertensive WT mice, the contractile response was essentially abolished in MA segments isolated from hypertensive LPP-KO mice (Figure 9b,d).

### 3.10. Distensibility of MA Segments Isolated from LPP-Deficient Mice Is Impaired

**Figure 10:**
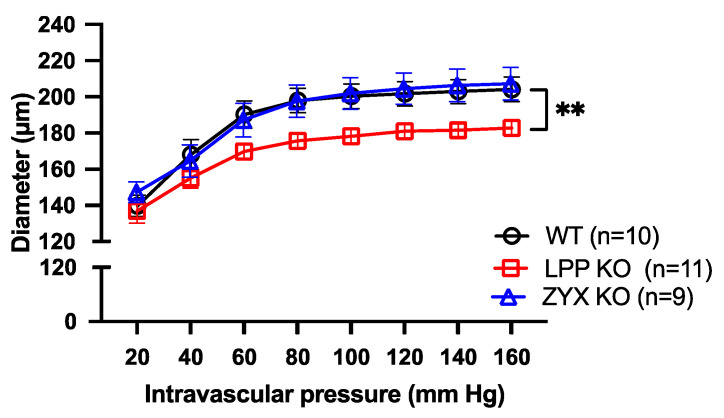
Passive pressure–diameter curves of 3rd-order MA segments isolated from WT, LPP-KO or ZYX-KO mice. The curves were generated in Ca^2+^-free physiological saline solution in the presence of EGTA. The number of isolated perfused segments derived from individual mice is indicated on the graph. ** *p* ˂ 0.01 as indicated.

Figure 10 shows the passive pressure–diameter (p-D) curves of isolated perfused MA segments in the absence of extracellular calcium. While the MA segments derived from the 6M WT or ZYX-KO mice revealed a rather similar p-D relationship to the p-D curve, reaching a plateau at about 80 mm Hg, the distension capacity of the pressurized MA segments derived from the age-matched LPP-KO mice was significantly smaller. There was a significant difference in the distention index between the MA segments derived from the WT as compared to LPP-KO mice (1.45 ± 0.02, n = 10 vs. 1.33 ± 0.04, n = 11, *p* = 0.0105) but not for the MA segments isolated from the ZYX-KO mice (1.39 ± 0.02, n = 9, *p* = 0.2902).

### 3.11. Thickness of the Vascular Wall in LPP-KO Mice

**Figure 11:**
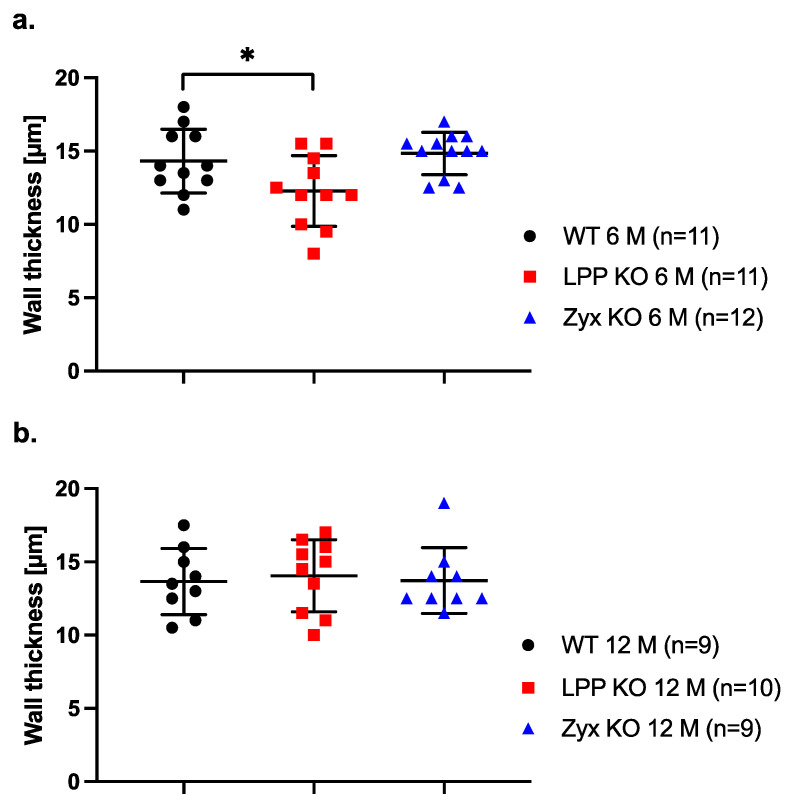
Passive wall thickness of 3rd-order MA segments isolated from (**a**) 6- and (**b**) 12-month-old WT, LPP-KO or ZYX-KO mice. Wall thickness was determined at 80 mm Hg in Ca^2+^-free physiological saline solution in the presence of EGTA (passive distention). The number of isolated perfused segments derived from individual mice is indicated on the graph. * *p* ˂ 0.05 as indicated.

We next asked whether the activated synthetic phenotype of LPP-KO VSMCs in culture is reflected by medial hyperplasia and thus an increased thickness of the vessel wall in MA segments isolated from LPP-KO mice. Such changes would have an impact on wall stress and hence affect the functional responses of the blood vessel to increased (blood) pressure. We therefore measured the thickness of the MA wall at 80 mm Hg. In MA segments isolated from 6-month-old mice, the thickness of the vessel wall was significantly smaller in LPP-KO mice as compared to their WT counterparts (Figure 11a). The thickness of the vessel wall of MA segments derived from the ZYX-KO mice was not different from that of the WT mice (Figure 11a). In MA segments derived from 12-month-old mice, there was no difference in wall thickness between the LPP-KO, WT or ZYX-KO mice (Figure 11b).

We also measured the distensibility of segments of the common carotid artery, a conduit artery with high values for its elastic properties. In contrast to the differences in the distensibility of the isolated perfused MA segments derived from WT and LPP-KO mice, we did not observe such differences in distensibility for carotid artery segments isolated from these animals (cf. Figure S7; distention index 1.54 ± 0.10, n = 6 vs. 1.65 ± 0.05, n = 6, *p* = 0.2338). The thickness of the pressurized carotid artery wall at 80 mm Hg was smaller in LPP-KO as compared to that of age-matched WT mice (37.4 ± 3.5, n = 5 vs. 44.9 ± 1.9 µm, n = 9, *p* = 0.0570), but this difference did not have statistical significance.

### 3.12. Age-Dependent Changes in LPP Expression in Mouse Blood Vessels

To examine whether there are any age-dependent changes with regard to LPP expression in arterial blood vessels, we employed Western blot analysis of isolated femoral artery segments. Figure S8 shows that the expression of LPP does not change with age in the femoral artery of the WT C57BL/6 mice. In contrast, LPP expression declines with age in the ZXX-KO mice. While the femoral artery segments isolated from the 12-month-old ZYX-KO mice revealed a slightly lower abundance of LPP as compared to that of the segments derived from their 6-month-old counterparts, the LPP protein levels dropped significantly in the femoral artery segments of the 18-month-old ZYX-KO mice.

## 4. Discussion

The adaptations of VSMCs to biomechanical stress require the systemic coordination of mechanosensors and their downstream signalling pathways. Previous studies have established LPP as an SMC-specific protein that localizes to FAs in which it is involved in the regulation of cell signalling, actin cytoskeleton organisation and cell migration [12,13,25]. These critical features prompted us to propose that LPP is an important coordinator of the cellular response to biomechanical stress in VSMCs.

To our knowledge, this is the first study describing the phenotype of VSMCs that lack a functional LPP protein. It reveals that these cells proliferate faster than their wildtype counterparts, and in a 3D environment, they also exhibit a greater degree of directed migration, suggesting that LPP plays a role in the phenotypic regulation of VSMCs by promoting the expression of genes and pathways associated with the quiescent contractile phenotype. In response to stretching, LPP redistributed towards stress fibres and the nucleus. This finding is in accordance with previous studies showing the ability of LPP to shuttle to the nucleus and enable activation of transcription [11].

Based on our studies, LPP in VSMCs may play a similar role as a mechanotransducer as its nearest relative zyxin, which in endothelial cells, rapidly translocates to the nucleus, acting as a mechanosensitive transcription factor [8]. In human and murine cultured VSMCs, zyxin promotes their quiescent contractile phenotype via the activation of the RhoA-myocardin-related transcription factor/serum response factor (MRTF/SRF) axis. The role of zyxin in VSMC phenotypic regulation is further reinforced by data showing that zyxin-deficient VSMCs in vitro proliferate and migrate faster [14]. Moreover, the overexpression of LPP in zyxin-deficient VSMCs reverts their activated synthetic phenotype to the quiescent contractile state [14]. This corresponds to our current observation: the overexpression of zyxin in the LPP-deficient VSMCs compensated for the lack of LPP by completely reverting their growth-promoting and pro-migratory phenotype. While LPP shares only 41% of its amino acid sequence identity with zyxin [11], both proteins harbour highly conserved domains, including a proline-rich N-terminal sequence with a nuclear export signal, VASP and α-actinin binding sites to interact both with the FAs and the cortical actin cytoskeleton. In addition, LPP and zyxin share three LIM domains within the C-terminal region that likely account for their ability to compensate for one another. This may also hold true for their mutual activation of the RhoA-MRTF-A/SRF axis that promotes, e.g., the expression of gene products, which are widely regarded as essential to maintain the quiescent contractile VSMC phenotype [26]. In this context, our spheroid contraction assay also shows that LPP-deficient spheroids respond with attenuated contraction to potassium chloride, pointing to the contractile deficit of these cells.

It has been established that cytoskeletal reorganization and actin dynamics directly affect MRTF-A/SRF signalling in myocytes [27]. The equilibrium between globular G actin and polymerized F actin determines not only the contractile state of the muscle cell and its motility, but also the expression of SRF-dependent genes. Thus, the higher concentration of G actin prevents MRTF-A translocation to the nucleus and the subsequent transcription of SRF-dependent genes supporting the contractile phenotype [28]. This effect of the actin cytoskeleton’s dynamics on the VSMC phenotype has also been demonstrated in vivo, in which the inhibition of stress fibre assembly by Rho A kinase inhibition prevents the proper differentiation of VSMCs and blood vessel formation due to the inhibition of the expression of SRF-dependent genes [29]. Since LPP interacts with VASP and α-actinin at the FAs thereby affecting actin polymerization dynamics, it may directly regulate MRTF-A localization and its targeting to the nucleus. This would explain why the lack of LPP in VSMCs not only diminishes the dynamic range of actin polymerization, resulting in an attenuated contraction/dilation of the LPP-deficient VSMC spheroids that we have observed, but also supports the growth-promoting and pro-migratory behaviour of these cells through its presumably indirect effect on VSMC-specific mechanosensitive gene expression.

The finding that LPP can be detected only in the media but not in the endothelium of isolated blood vessels from adult mice strongly supports the VSMC-specific role of LPP. Further, RNA sequencing data on brain and lung vasculature suggest that there is also a differential expression of LPP along the vascular tree (arterial VSMCs > arteriolar VSMCs > venous VSMCs, http://betsholtzlab.org/VascularSingleCells/database.html (accessed on 8 February 2023)). In this context, it is interesting to note that the contractile response to increases in perfusion pressure is attenuated in resistance-sized arteries isolated from LPP-deficient mice with increasing age while their contractile capacity for phenylephrine, potassium chloride (i.e., depolarisation) or ET-1 remains unchanged. Since LPP is highly abundant at FAs, one explanation for our results could be that the absence of LPP slows down the kinetics by which the increase in wall tension/stretching is transduced to the actin cytoskeleton and beyond.

LPP-KO mouse mesenteric artery exhibited an attenuated passive distention capacity that could also contribute to their lower myogenic responses because the relative constriction becomes greater with the increased diameter at baseline. In addition to structural changes in the ECM in the arterial vessel wall, studies indicate that chronic changes in the biomechanical properties of VSMCs, such as alterations in actin dynamics, lead to the stiffening of the VSMCs themselves, thereby affecting the biomechanical properties of the blood vessel, too [30]. Interestingly, only the mesenteric artery segments isolated from aged LPP-KO mice exhibited an impaired myogenic response. While there is some evidence that advanced age leads to an attenuated myogenic responsive in small arteries and arterioles of the mesentery [31], many studies demonstrate this effect of aging also in other vascular beds (as summarized in [32]). Accordingly, our data suggest that LPP as an important regulator of actin dynamics helps properly organize the actin cytoskeleton and maintain the phenotype of the medial VSMCs, thereby sustaining the elastic properties of the blood vessel wall.

In addition, we considered other potential mechanisms contributing to the attenuated myogenic tone in the MA segments isolated from LPP-deficient mice. In view of the increased MMP activity in the cultured LPP-deficient VSMCs, we hypothesized that this could influence the functional response of the isolated mesenteric artery segments. While the multiple roles of matrix metalloproteinases, including the remodelling of the ECM, VSMC proliferation, migration and differentiation have been well recognized [33], they also exhibit acute effects by inhibiting calcium entry pathways and vasoconstriction [34,35]. In our study, the general MMP inhibitor GM6001 enhanced the myogenic response of mesenteric artery segments isolated from LPP-KO but not WT mice, suggesting that the increase in MMP activity in the VSMCs of these blood vessels mitigates the myogenic response, possibly through the inhibition of calcium influx as previously reported [34,35]. Thus, the activated synthetic phenotype of the LPP-KO VSMCs may affect the vasomotor tone not only by altering the structural characteristics of these blood vessels, but also more acutely by modifying their processing and/or release of vasoactive mediators.

Moreover, our finding that the myogenic response of MA segments isolated from mice made hypertensive (by using the DOCA-salt model) is largely mediated by the non-selective cation channel TRPC3 is in agreement with previous studies showing that both the expression and function of this cation channel is increased in VSMCs isolated from the mesenteric vascular bed of spontaneously hypertensive mice [36]. TRPC3 and TRPC 6, which can form both homo- and heterotetrameric complexes, are known to mediate stretch-induced cation influx into VSMCs, causing their depolarization followed by the activation of voltage-dependent LTCC, influx of extracellular calcium and subsequent constriction [37]. While Pyr3 was first described as a selective blocker of TRPC3 [24], there are reports that it can also inhibit TRPC6-dependent cation influx as well as cation currents carried by TRPC3/TRPC6 heterotetramers [36]. Our finding that Pyr3 differentially blocked the myogenic response of the MA segments isolated from hypertensive WT and LPP-KO mice points to possible differences in composition of the TRPC channels involved.

Even though our in vitro studies suggest that LPP-KO VSMCs proliferate and migrate faster than their WT counterparts, we did not observe any signs of hypertrophy (or hyperplasia) in the conduit or resistance-sized arteries isolated from the LPP-KO mice. Because VSMCs in culture start to dedifferentiate within a few days after isolation [1], it is likely that in the absence of LPP, which promotes proper cytoskeletal reorganization and actin dynamics as well as the expression of genes encoding contractile proteins, the process of dedifferentiation is accelerated or intensified.

Of the 63 publications on this LIM-domain protein to date, more than half suggest a role for LPP in tumour cell migration, invasion and metastasis. This association is reasonable when considering that LPP promotes mesenchymal cell/fibroblast migration, localizes to cellular adhesions and even promotes invadopodia formation [38]. However, these tumorigenic and metastasis-promoting functions require LPP, while in our study, it is the loss of LPP that promotes the phenotypic dedifferentiation of VSMCs characterized by increased migration, proliferation and reduced contractile properties. Moreover, according to own analyses of the microarray data from a publicly available database, reduced LPP abundance may be related to arterial aneurysm formation and rupture in humans, another maladaptive remodelling process affecting extracellular matrix composition and stability in conduit arterial blood vessels that may originate from these cells. Thus, LPP and other Janus-faced mechanotransducers, e.g., of the YAP/TAZ signalling pathway, seem to be essential to maintaining these cells in the differentiated contractile state for normal vascular function.

## 5. Conclusions

Lipoma-preferred partner (LPP) is an LIM domain protein selectively expressed by smooth muscle cells, and the current study emphasizes its unique role in the vasculature. To our knowledge, this study is the first to characterize the ex vivo phenotype of LPP-deficient vascular smooth muscle cells as well as the functional properties of blood vessels lacking an intact LPP protein. Our findings provide the first insight into the complex interplay of this novel mechanotransducer in outside-in signalling from the extracellular matrix to the nucleus of both cultured and native vascular smooth muscle cells in response to increased stretching and hence wall tension. Most notably, they reveal a critical role for LPP in maintaining the quiescent contractile phenotype of vascular smooth muscle cells in the face of a chronic rise in blood pressure as it occurs, e.g., in arterial hypertension.

## Data Availability

The datasets generated during the current study are not publicly available but are available from the corresponding author on reasonable request.

## Supplementary Materials

The following supporting information can be downloaded at: https://www.mdpi.com/article/10.3390/cells12182315/s1, Figure S1: BrdU incorporation assay was performed to compare the proliferation rate of WT vs LPP knockout (LPP KO) vascular smooth muscle cells (VSMC) isolated from WT and LPP-KO mouse aorta; Figure S2: Overexpression of zyxin in the LPP KO VSMC transfected with a zyxin expression construct raises the abundance of zyxin in the VSMC approximately 3-fold; Figure S3: LPP KO VSMC spheroids exhibit a greater gelatinase activity, detected by the increased fluorescence emanating due to DQ-gelatine digestion, as compared to their WT counterparts; Figure S4: (a) While potassium chloride (KCl; 60 mM) produced a contraction of the three-dimensional (3D) VSMC spheroids (isolated from the aorta of WT mice) grown on top of a collagen gel this contraction was inhibited (presented as relative reduction of the surface area of the 3D VSMC spheroid covering the collagen gel) but not abolished by pre-treatment with the voltage-dependent L-type Ca^2+^ channel blocker nifedipine (Nif; 10 µM). (b) In contrast to KCl, norepinephrine (NE) produced a relaxation of the 3D VSMC spheroids, which was abolished by pre-incubation with propranolol (10 μM); Figure S5: Meta-analysis of single cell RNA sequencing data of different vascular cells isolated from the pulmonary and brain microcirculation shows that LPP is highly enriched in the VSMC as compared to VSMC-like pericytes or microvascular endothelial cells; Figure S6: Myogenic response of 3rd and 4th order mesenteric artery (MA) segments isolated from DOCA-salt treated hypertensive LPP KO mice; Figure S7: Passive pressure-diameter curves of common carotid artery segments isolated from 4 to 6-month-old WT and LPP KO mice; Figure S8: Abundance of LPP in the femoral artery of zyxin (ZYX) knockout mice changes in an age-dependent manner.
