# Supplementary material for: Characterisation of Lipoma-Preferred Partner as a Novel Mechanotransducer in Vascular Smooth Muscle Cells
Source: Cells. 2023 Sep 19;12(18):2315. doi: 10.3390/cells12182315 (PMC10529303; doi:10.3390/cells12182315)
Supplement: Supplementary file 1 [file cells-12-02315-s001.zip › cells-2564800-supplementary-2_final version.pptx]

## Slide 1
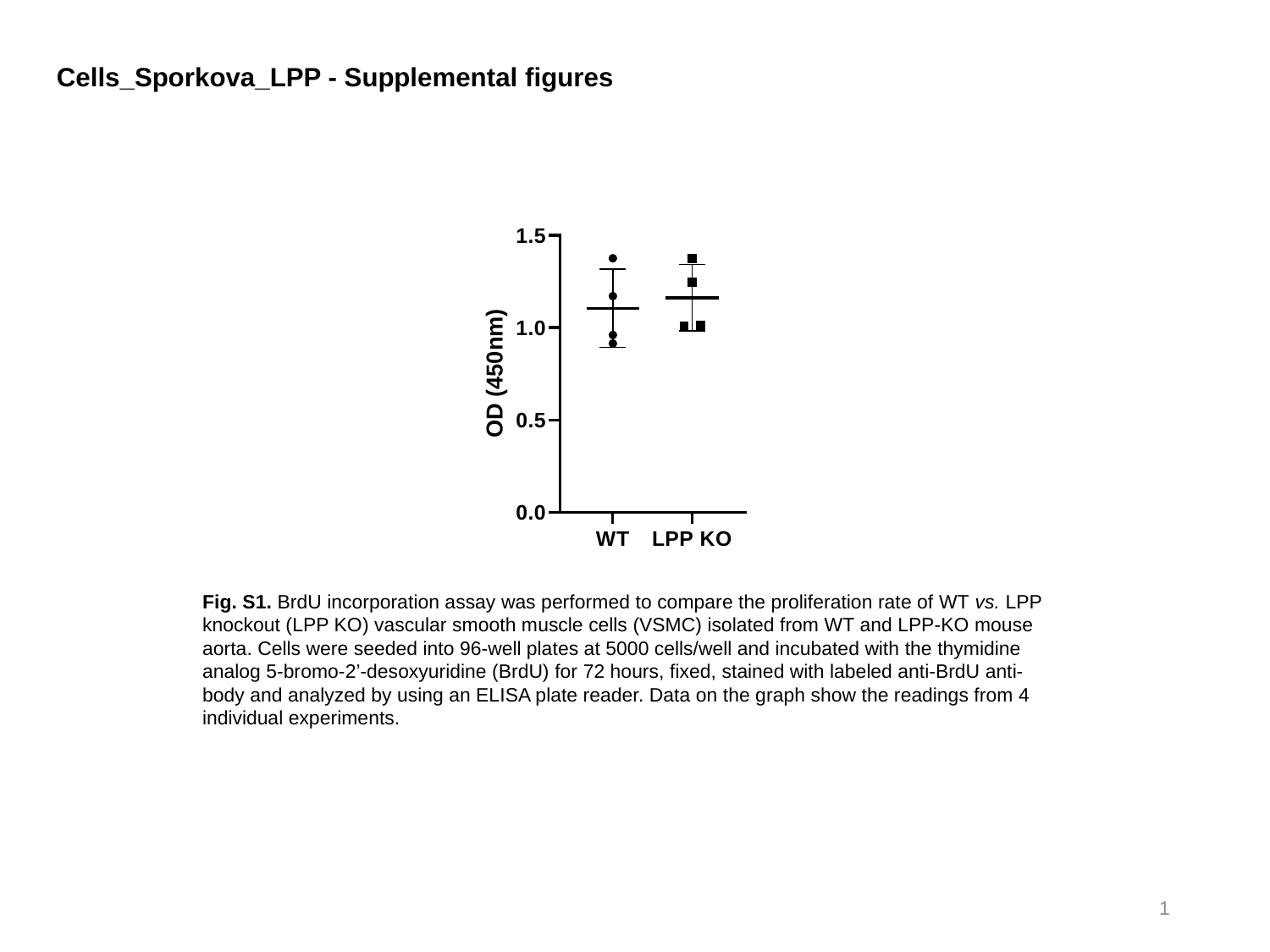

Cells_Sporkova_LPP - Supplemental figures
Fig. S1. BrdU incorporation assay was performed to compare the proliferation rate of WT vs. LPP knockout (LPP KO) vascular smooth muscle cells (VSMC) isolated from WT and LPP-KO mouse aorta. Cells were seeded into 96-well plates at 5000 cells/well and incubated with the thymidine analog 5-bromo-2’-desoxyuridine (BrdU) for 72 hours, fixed, stained with labeled anti-BrdU anti-body and analyzed by using an ELISA plate reader. Data on the graph show the readings from 4 individual experiments.
1

## Slide 2
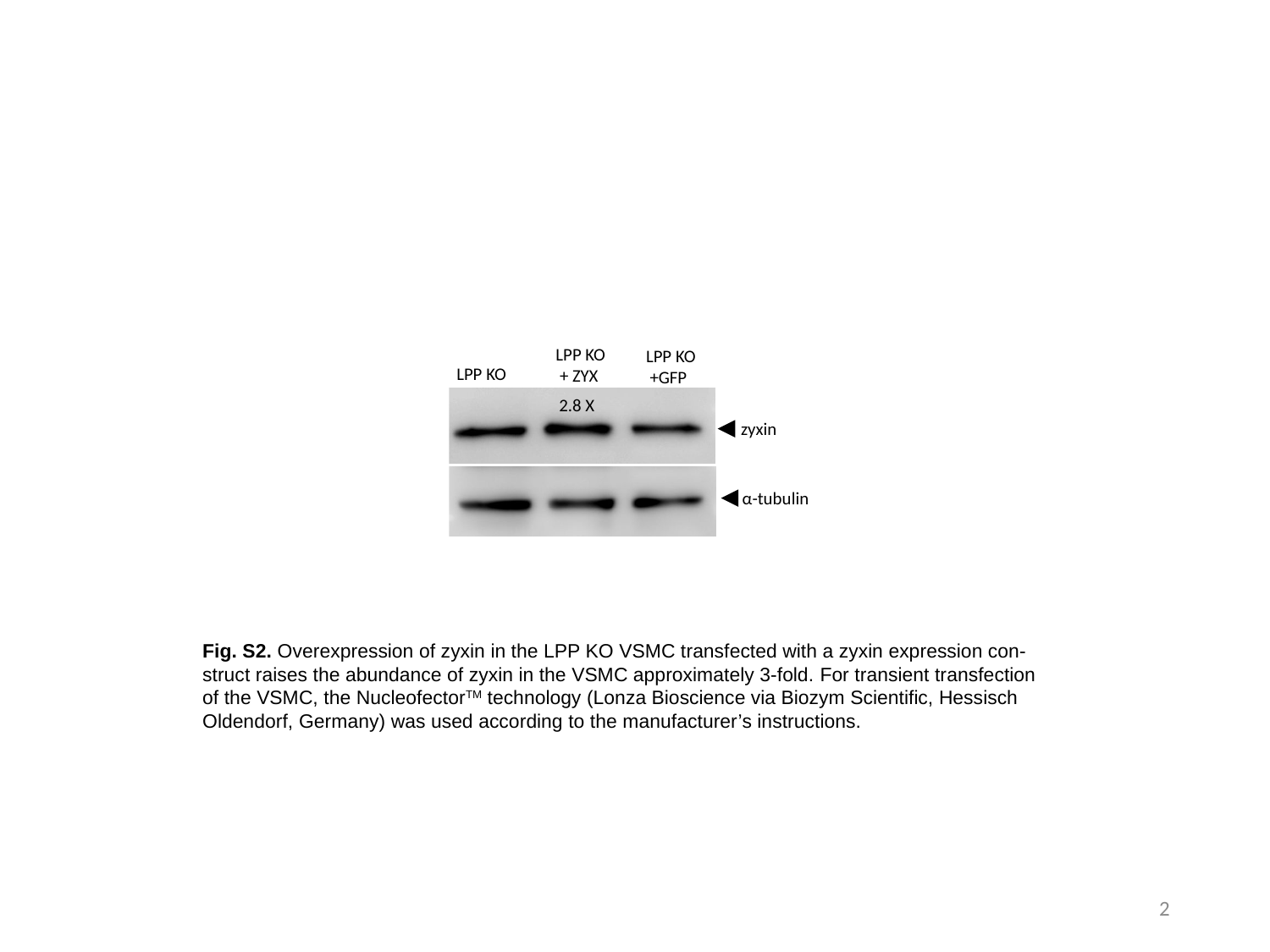

LPP KO
 + ZYX
LPP KO
 +GFP
LPP KO
2.8 X
zyxin
α-tubulin
Fig. S2. Overexpression of zyxin in the LPP KO VSMC transfected with a zyxin expression con-struct raises the abundance of zyxin in the VSMC approximately 3-fold. For transient transfection of the VSMC, the NucleofectorTM technology (Lonza Bioscience via Biozym Scientific, Hessisch Oldendorf, Germany) was used according to the manufacturer’s instructions.
2

## Slide 3
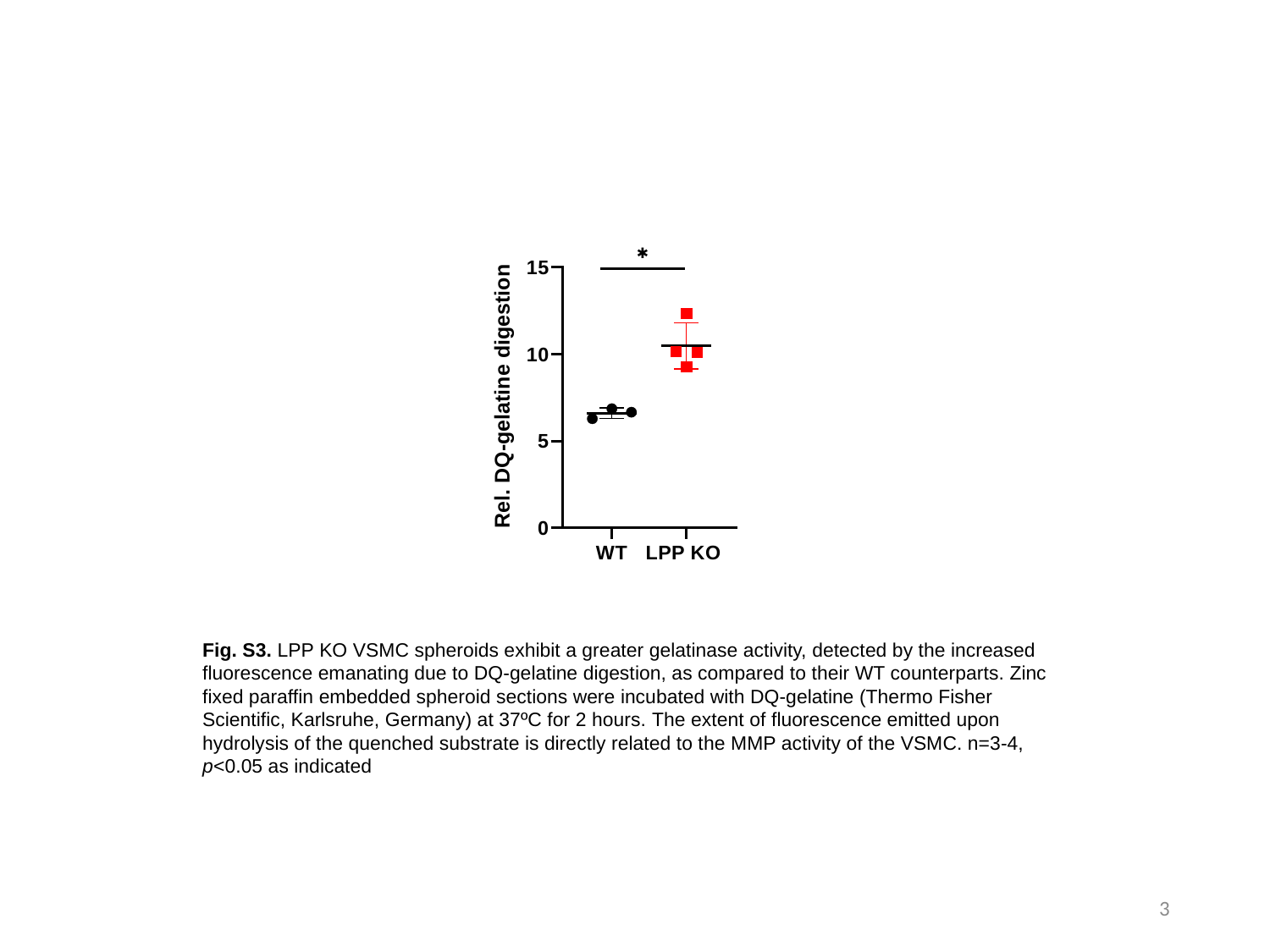

Rel. DQ-gelatine digestion
Fig. S3. LPP KO VSMC spheroids exhibit a greater gelatinase activity, detected by the increased fluorescence emanating due to DQ-gelatine digestion, as compared to their WT counterparts. Zinc fixed paraffin embedded spheroid sections were incubated with DQ-gelatine (Thermo Fisher Scientific, Karlsruhe, Germany) at 37ºC for 2 hours. The extent of fluorescence emitted upon hydrolysis of the quenched substrate is directly related to the MMP activity of the VSMC. n=3-4, p<0.05 as indicated
3

## Slide 4
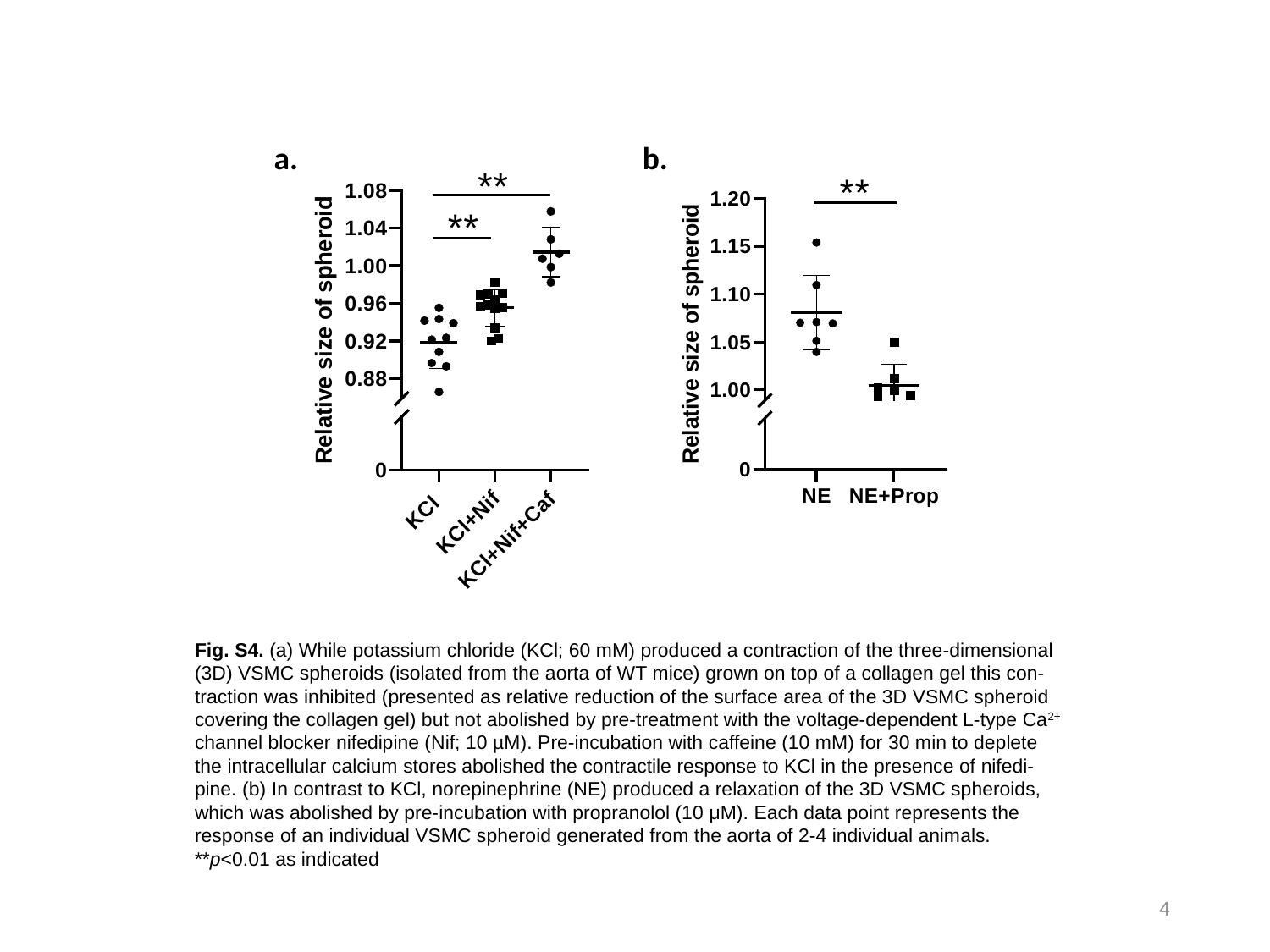

b..
a..
Fig. S4. (a) While potassium chloride (KCl; 60 mM) produced a contraction of the three-dimensional (3D) VSMC spheroids (isolated from the aorta of WT mice) grown on top of a collagen gel this con-traction was inhibited (presented as relative reduction of the surface area of the 3D VSMC spheroid covering the collagen gel) but not abolished by pre-treatment with the voltage-dependent L-type Ca2+ channel blocker nifedipine (Nif; 10 µM). Pre-incubation with caffeine (10 mM) for 30 min to deplete the intracellular calcium stores abolished the contractile response to KCl in the presence of nifedi-pine. (b) In contrast to KCl, norepinephrine (NE) produced a relaxation of the 3D VSMC spheroids, which was abolished by pre-incubation with propranolol (10 μM). Each data point represents the response of an individual VSMC spheroid generated from the aorta of 2-4 individual animals. **p˂0.01 as indicated
4

## Slide 5
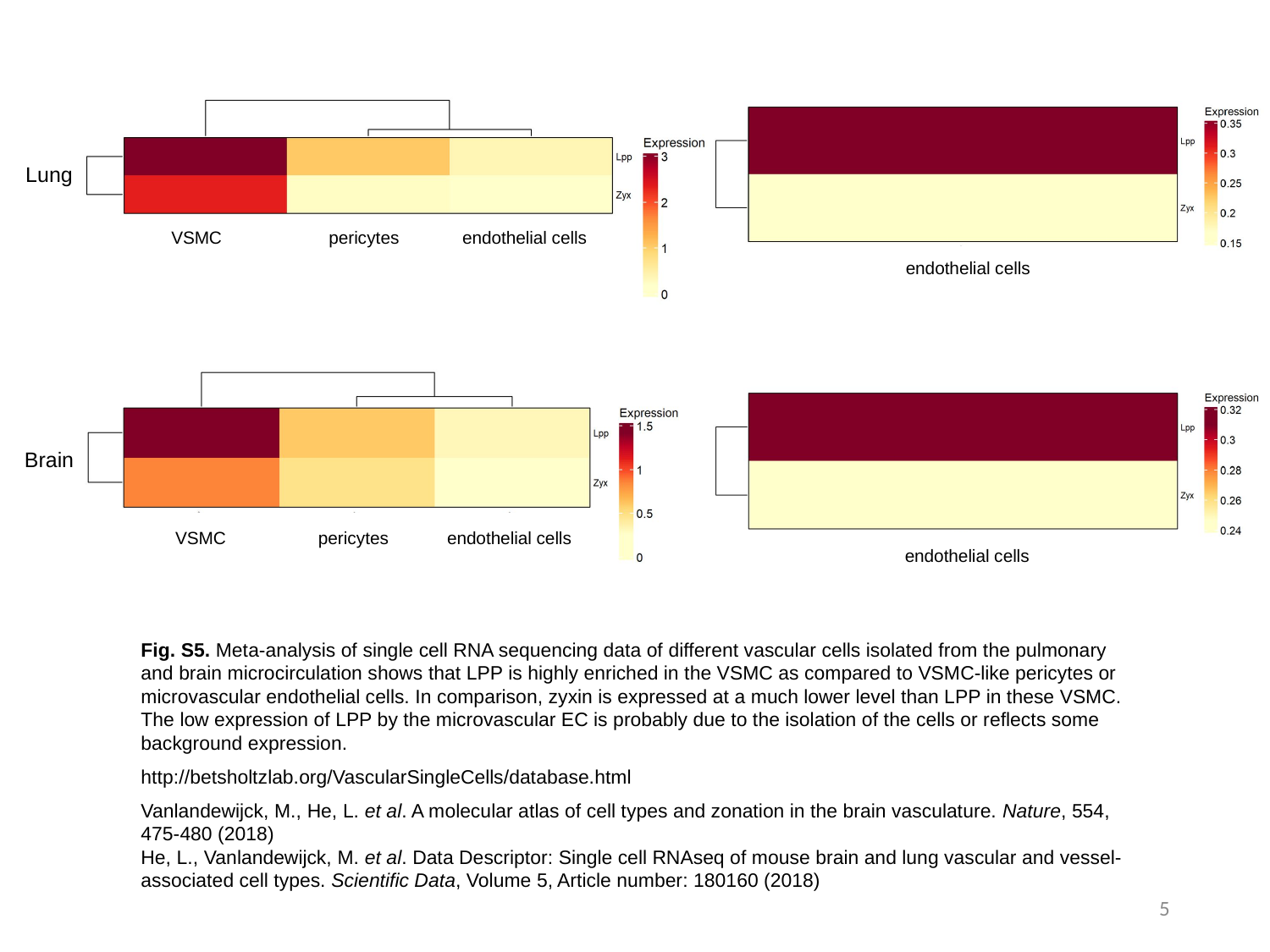

Lung
Brain
VSMC pericytes endothelial cells
endothelial cells
VSMC pericytes endothelial cells
endothelial cells
Fig. S5. Meta-analysis of single cell RNA sequencing data of different vascular cells isolated from the pulmonary and brain microcirculation shows that LPP is highly enriched in the VSMC as compared to VSMC-like pericytes or microvascular endothelial cells. In comparison, zyxin is expressed at a much lower level than LPP in these VSMC. The low expression of LPP by the microvascular EC is probably due to the isolation of the cells or reflects some background expression.
http://betsholtzlab.org/VascularSingleCells/database.html
Vanlandewijck, M., He, L. et al. A molecular atlas of cell types and zonation in the brain vasculature. Nature, 554, 475-480 (2018)
He, L., Vanlandewijck, M. et al. Data Descriptor: Single cell RNAseq of mouse brain and lung vascular and vessel-associated cell types. Scientific Data, Volume 5, Article number: 180160 (2018)
5

## Slide 6
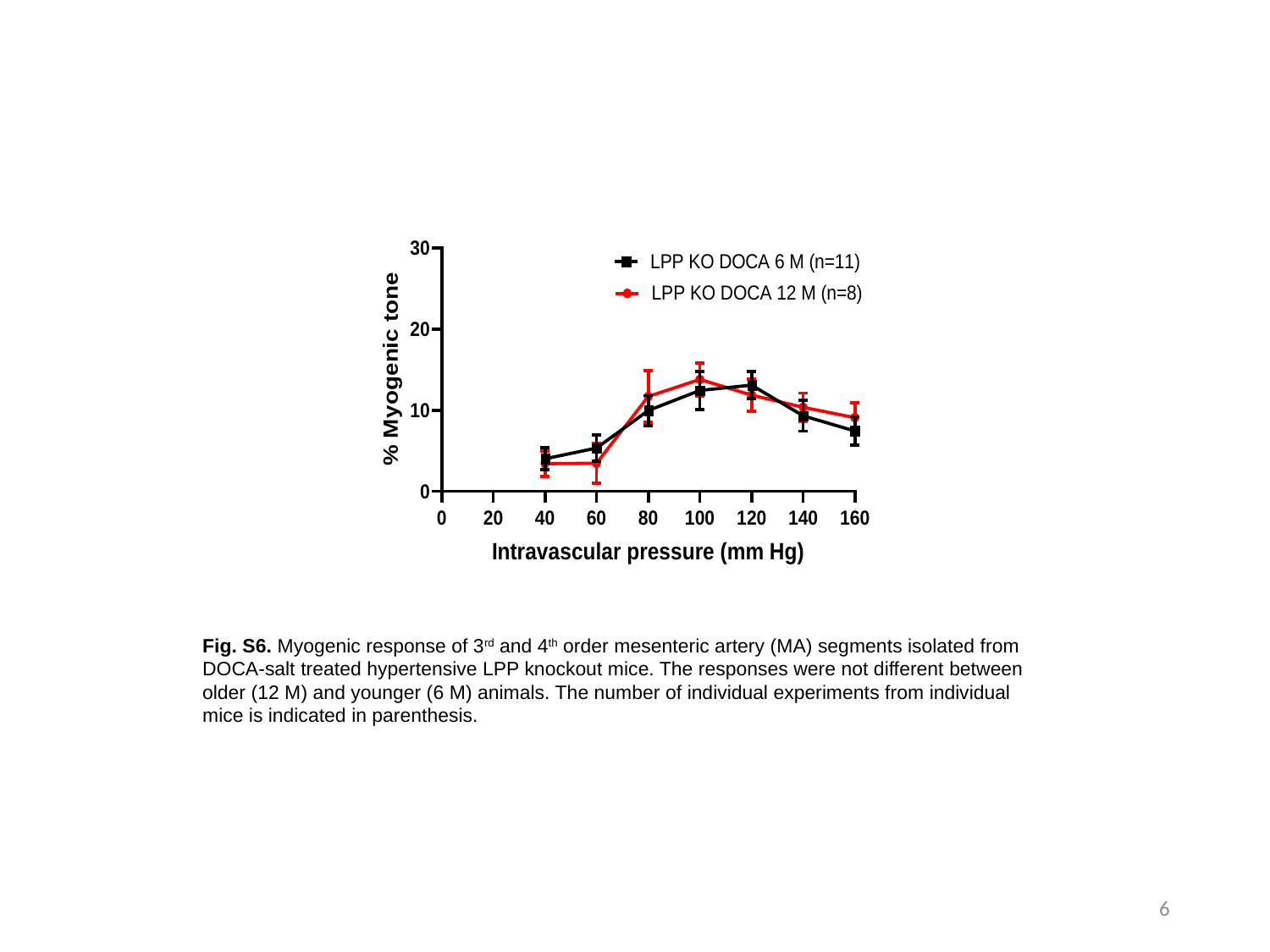

Fig. S6. Myogenic response of 3rd and 4th order mesenteric artery (MA) segments isolated from DOCA-salt treated hypertensive LPP knockout mice. The responses were not different between older (12 M) and younger (6 M) animals. The number of individual experiments from individual mice is indicated in parenthesis.
6

## Slide 7
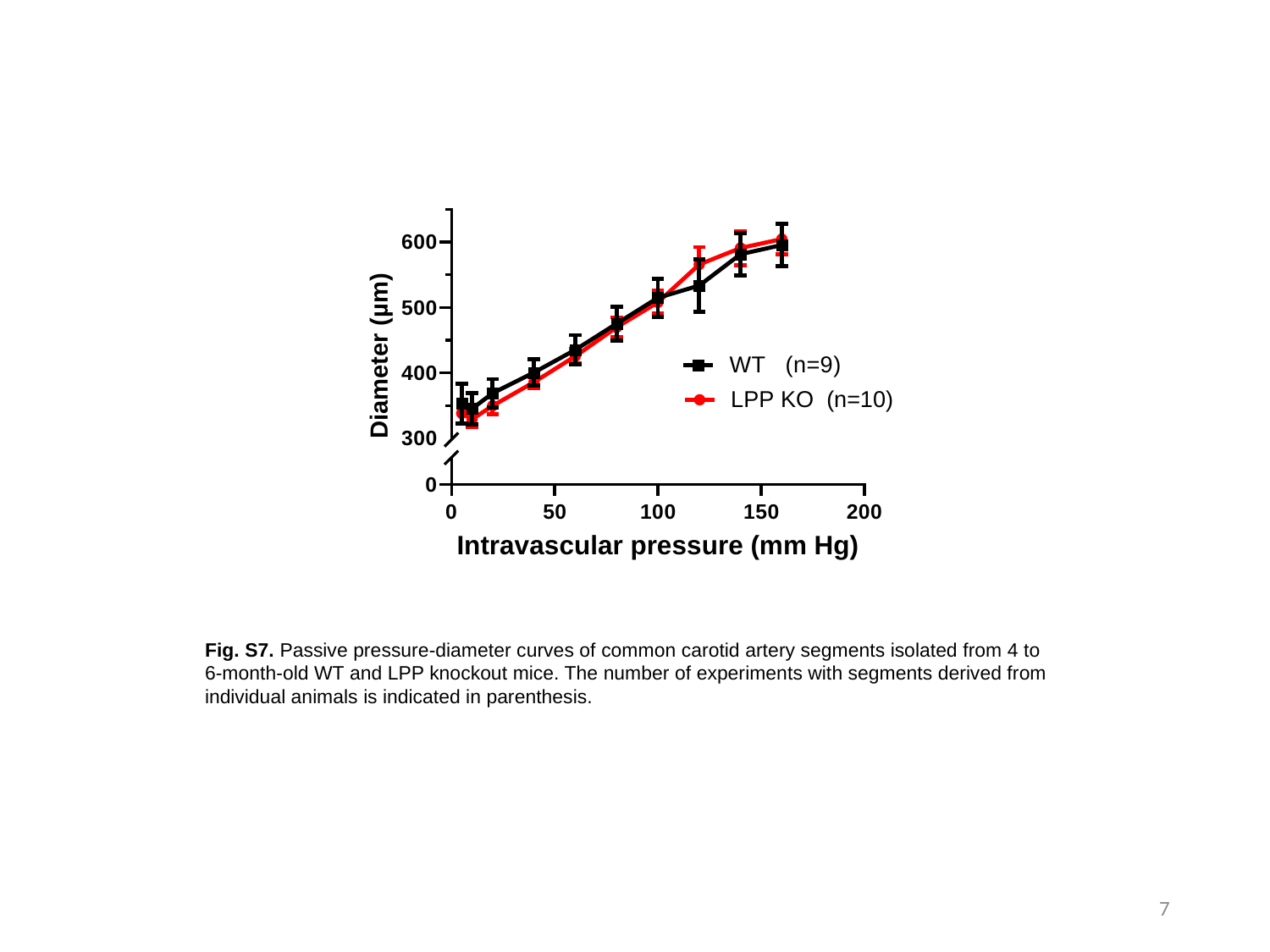

Diameter (µm)
Fig. S7. Passive pressure-diameter curves of common carotid artery segments isolated from 4 to 6-month-old WT and LPP knockout mice. The number of experiments with segments derived from individual animals is indicated in parenthesis.
7

## Slide 8
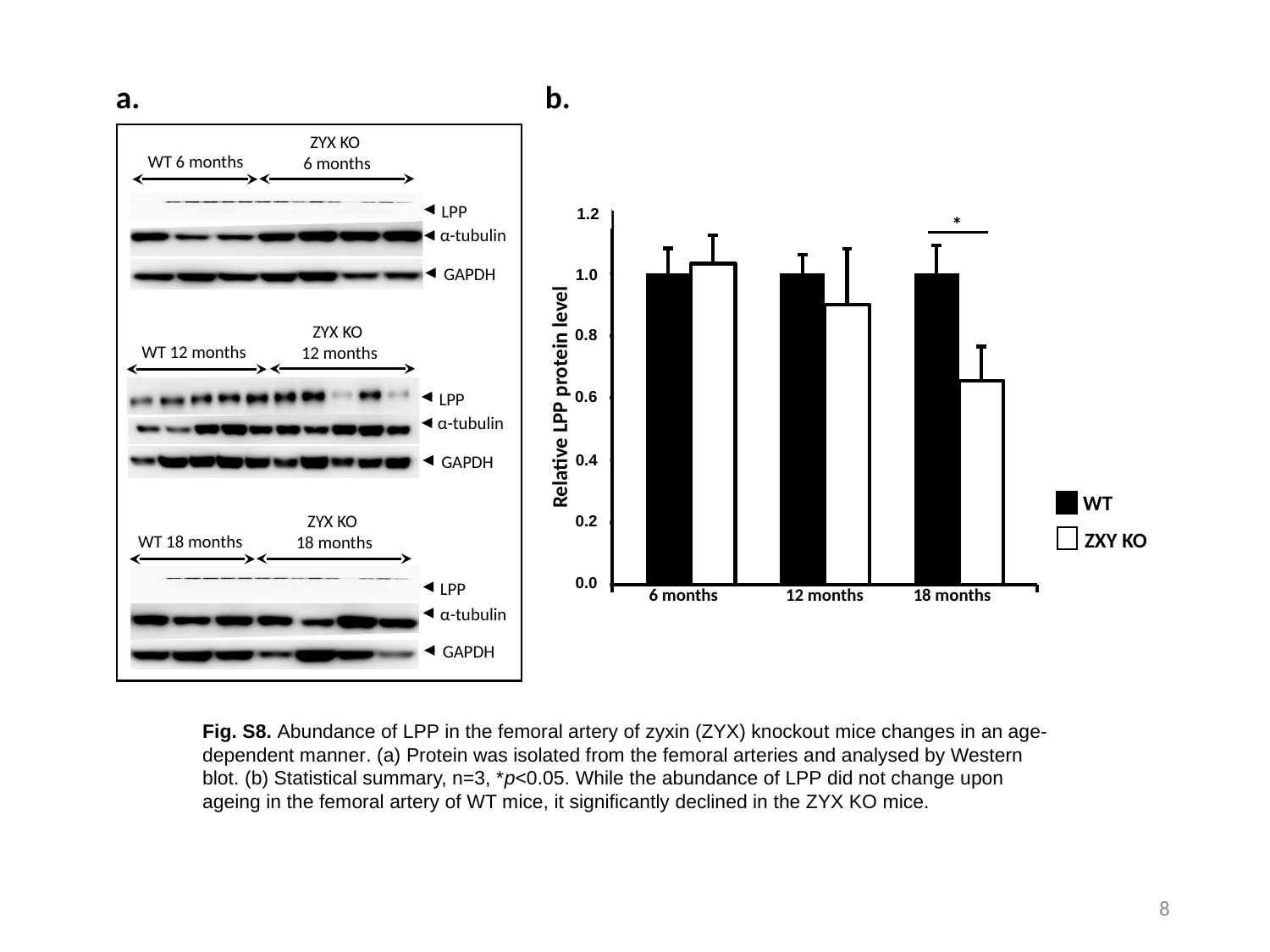

b..
a..
ZYX KO
 6 months
WT 6 months
LPP
α-tubulin
GAPDH
ZYX KO
 12 months
WT 12 months
LPP
α-tubulin
GAPDH
ZYX KO
 18 months
WT 18 months
LPP
α-tubulin
GAPDH
### Chart
| Category | WT 6m | Zyx ko 6m | | WT 12 m | Zyx ko 12m | | WT 18 m | Zyx ko 18 m |
|---|---|---|---|---|---|---|---|---|1.2
*
1.0
G.
0.8
0.6
Relative LPP protein level
0.4
WT
ZXY KO
0.2
0.0
6 months
12 months
18 months
Fig. S8. Abundance of LPP in the femoral artery of zyxin (ZYX) knockout mice changes in an age-dependent manner. (a) Protein was isolated from the femoral arteries and analysed by Western blot. (b) Statistical summary, n=3, *p<0.05. While the abundance of LPP did not change upon ageing in the femoral artery of WT mice, it significantly declined in the ZYX KO mice.
8
